# Soft-robotic green sea turtle (*Chelonia mydas*) developed to replace animal experimentation provides new insight into their propulsive strategies

**DOI:** 10.1038/s41598-023-37904-5

**Published:** 2023-07-25

**Authors:** Nick van der Geest, Lorenzo Garcia, Fraser Borret, Roy Nates, Alberto Gonzalez

**Affiliations:** BioDesign Lab, Auckland University of Technology, Auckland, 1010 New Zealand

**Keywords:** Biomechanics, Mechanical engineering

## Abstract

Green sea turtles (*Chelonia mydas*) can swim up to 50 km per day while only consuming seagrass or microalgae. How the animal accomplishes this vast journey on such low energy intake points to the effectiveness of their swimming technique and is a testament to the power of evolution. Understanding the green sea turtle's ability to accomplish these journeys requires insight into their propulsive strategies. Conducting animal testing to uncover their propulsive strategies brings significant challenges: firstly, the ethical issues of conducting experiments on an endangered animal, and secondly, the animal may not even swim with its regular routine during the experiments. In this work, we develop a new soft-robotic sea turtle that reproduces the real animal's form and function to provide biomechanical insights without the need for invasive experimentation. We found that the green sea turtle may only produce propulsion for approximately 30% of the limb beat cycle, with the remaining 70% exploiting a power-preserving low-drag glide. Due to the animal's large mass and relatively low drag coefficient, losses in swim speed are minimal during the gliding stage. These findings may lead to the creation of a new generation of robotic systems for ocean exploration that use an optimised derivative of the sea turtle propulsive strategy.

## Introduction

**Figure 1:**
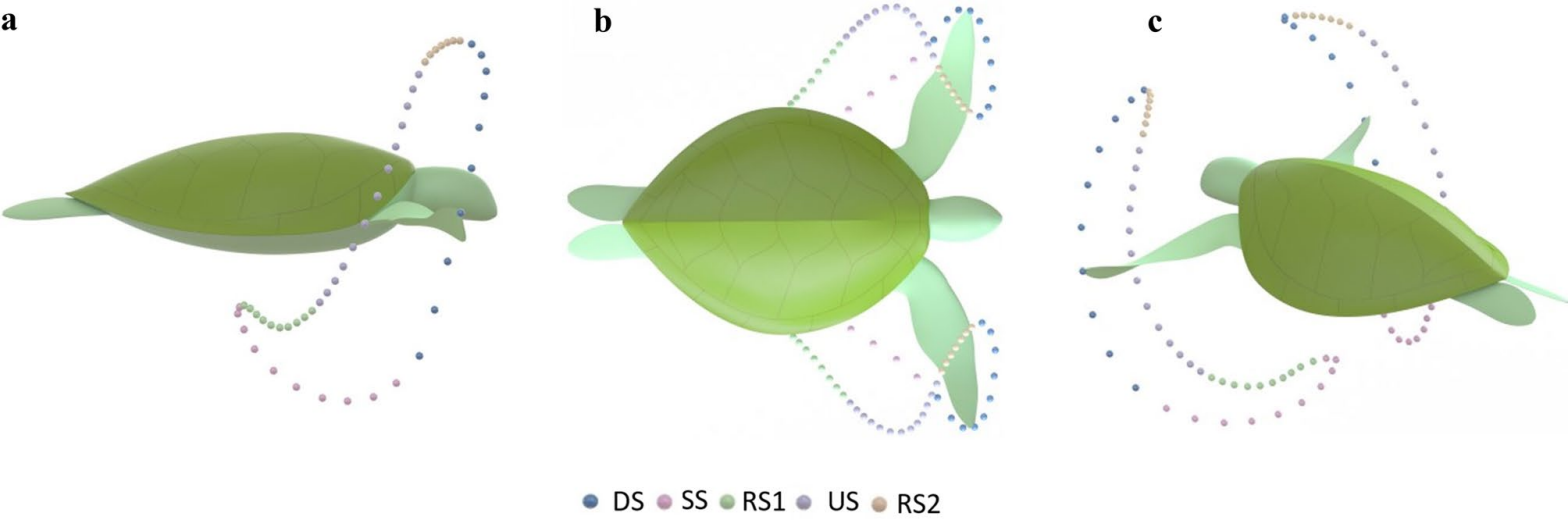
Five stages of the green sea turtle's swimming pattern as illustrated with coloured spheres^5^. (**a**) Swim pattern viewed from the sagittal plane (**b**) Swim pattern viewed from the Coronal plane. (**c**) Swim pattern viewed from a 3D viewpoint.

The migrations of green sea turtles (*Chelonia mydas*) have been shown to cover as far as 2342 km in only 47 days, the equivalent of travelling 50 km per day^1^. What makes this journey remarkable is the low-energy foods they consume, mainly seagrass and microalgae^2^.The biology of sea turtles dominates the literature, yet, research into the animal's propulsive performance has had little investigation, with most work simplifying their analysis to a rigid body with simplified kinematics^3,4^. Understanding the sea turtle's propulsion methods could potentially lead to the development of highly efficient robots for long-term underwater missions that take inspiration from the animal's locomotor patterns. However, gaining this understanding is of enormous complexity. Firstly, studying an endangered live animal brings tremendous inconvenience in obtaining ethical approvals^5^. Additionally, the animal may not even swim with its natural locomotor pattern when in captivity^5^. To overcome these limitations, researchers have tried to design and build animal-inspired robots to help comprehend and take advantage of unique animal characteristics^6–12^. Developing a robotic sea turtle could be the solution to uncovering the sea turtle's secrets in propulsion and energy consumption. Some robotic sea turtles have been created in the past^13–19^ however, all attempts have failed to entirely mimic the sea turtle's locomotor pattern and flexible limbs. In some cases, these attempts have only introduced two degrees of freedom systems^13,14,16^. It is well understood that sea turtles produce complex three degrees of freedom motion, demonstrating the shortfall in those studies to reproduce genuine kinematics. While other works have introduced three degrees of freedom^15,17,19,20^ the main goal in their works was not to try and truly mimic the sea turtle but demonstrate a novel turtle-inspired robotic technology. In recent work, an updated sea turtle locomotor description for the animal's general swimming routine is provided by van der Geest et al.^5^, which details the 3-dimensional flipper patterns, including the active twisting of the flipper. They found that wild green sea turtles had a different swimming pattern than previous studies on juveniles in capitivity^21^. Additionally, they outlined how the flipper's motion can be broken up into five stages consisting of: downstroke (DS), sweep stroke (SS), recovery stroke one (RS1), upstroke (US) and, recovery stroke two (RS2) (Fig. 1). During the green sea turtles general swimming routine it is understood green sea turtles produce an average limb beat cycle of about 0.23 Hz^5,22^ to swim an average speed of approximately 0.6 m/s^22–25^.

Developing a robotic sea turtle that can genuinely mimic a real animal brings two significant complexities: firstly, the flippers need to be a soft robotic device that can actively change shape to achieve optimum levels of wing twist while simultaneously supporting large flexural loads from the hydrodynamic forces. This is difficult to achieve with rigid robots that require stiff and multiple mechanisms leading to intricate assemblies to realize complex movements. Hence, soft robotic devices have been developed, as they can use simple deformations to achieve natural movements mimicking biological systems^26–28^. Current soft wing twisting technology has, in most cases, taken two forms: the first form produces actuation via shape memory alloys (SMA) or ionic polymer-metal composite (IPMC)^17,29,30^. However, as a creative approach, these solutions fail to produce large hydrodynamic forces when used in flapping wing conditions. The second form comes in solutions that include a soft material in the rigid robot, adding compliance to the system and allowing them to generate complex movements^6,10,31,32^. These solutions can support higher hydrodynamic loading at the expense of more complex design and manufacturing requirements. The second complexity to producing a realistic robotic sea turtle is that the overall form factor of the robot should look identical to that of the actual animal, thus making packaging of the required hardware within the robotic limbs a tedious and ambitious design challenge. In recent work by Yan et al.^18^, a turtle-inspired robot with variable stiffness hydrofoils describes a design that can vary the spanwise stiffness of the flippers with an assembly of small rigid bodies. Although it shows novel elements, it fails to realise an essential aspect of the sea turtle's flippers: the spanwise twist. The continuous smooth (one single body) twisting of the pectoral flippers is essential to ensure the correct angle of attack is produced along the flipper span to achieve maximum efficiency.

**Figure 2:**
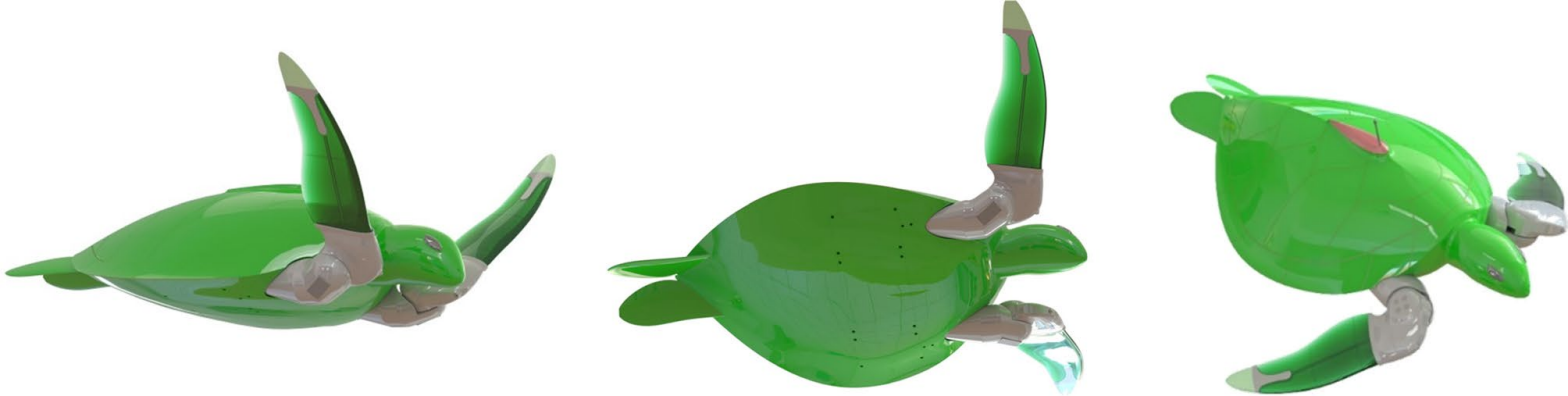
Final sea turtle robot design based on the green sea turtle (*Chelonia mydas*).

To address these challenges, we reverse-engineered the natural sea turtle into a robotic form to attempt and replicate the real turtle's form and function (Fig. 2 and Movie S1). To the best of our knowledge, this is the world's first sea turtle robot designed exclusively for replicating the natural animal locomotion. The goal of this robot is not to enhance or improve sea turtle locomotion but gain further insight into how this animal produces propulsion. This new robot design is equipped with soft robotic flippers and a rigid shell, made possible by taking advantage of modern additive manufacturing equipment. The design can reproduce the natural swimming patterns based on the natural form of the green sea turtle (*Chelonia mydas*) (Movie S1). The flipper design, consists of a variable stiffness (across the span) polyurethane rubber that is cast in situ over a central carbon fibre spar that is connected to an additively manufactured rigid flipper tip. The design can produce near perfect linear twist along its span up to the rigid zone as per the twisting motion of real green sea turtles^5^ (Movie S2) using only a single actuator. To determine the mechanical performance and endurance of the soft flipper design, bending and fatigue tests were performed. These results indicated that the design could support large flexural loads and operate robustly throughout its intended lifespan (Movie S3).

To reveal the propulsion performance, testing was performed within a freshwater tow tank to measure power consumption, thrust, drag and lift forces during the animal's regular swimming routine. Our findings show that the animal only produces thrust for approximately 1.4 s of the animal's 4.3-s flipper oscillation to produce an average swim speed of 0.602 m/s. During the remaining 2.9 s, the flippers go into a remarkable energy-saving recovery glide that does not substantially reduce the swim speed due to the animal's large mass and relatively low drag coefficient.

These findings advance our understanding of how sea turtles produce propulsion during their regular swimming routine. Thus, these findings may lead to new opportunities to optimise the findings for higher propulsive performance and efficiency to enhance the next generation of ocean exploration technologies.

## Results

### Sea turtle robot design

**Figure 3:**
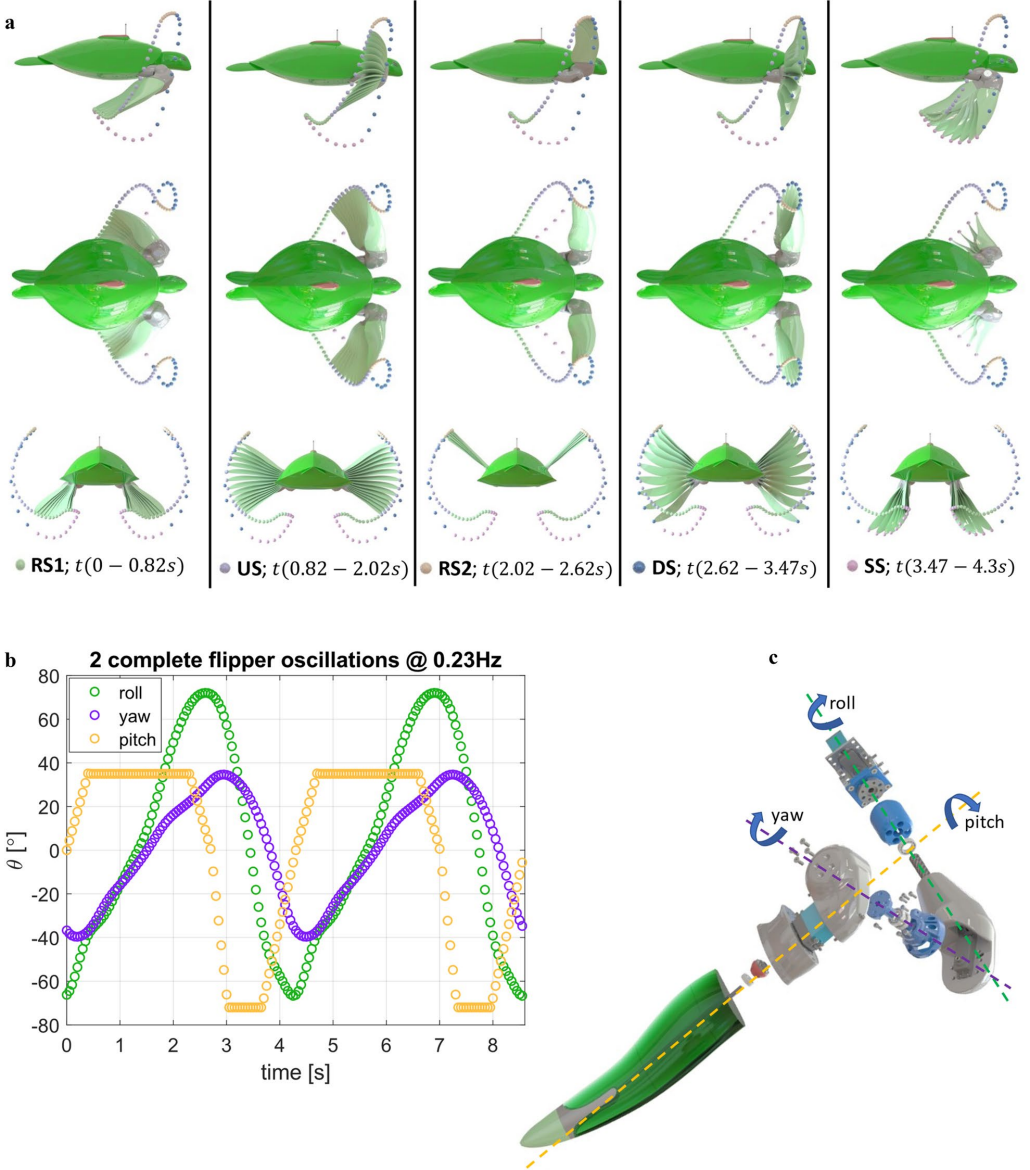
Sea turtle locomotion patterns. (**a**) Robot sea turtle assembly showing flipper position and twist @ 0.08 s time intervals in the five stages of the sea turtle locomotor pattern consisting of the downstroke (DS), sweep stroke (SS), recovery stroke one (RS1), upstroke (US), and recovery stroke two (RS2) as viewed from the sagittal, coronal and transverse planes. Time taken to complete each of the five stages is presented at the bottom of each column (**b**) Equations of motion in roll, yaw and pitch (Twist) plotted against time for two complete flipper cycles at a flapping frequency of 0.23 Hz. (**c**) Exploded parts view of the sea turtle robot limb showing the rotation axis locations and hardware.

Designing the robot to meet a real sea turtle's natural form and function brought with it many levels of complexity. Most of which come from packaging the required hardware into the organic surfaces that make up the turtle's form. The design process took an iterative approach by starting with a CAD model based on real sea turtles developed through multiplane extraction of video footage^5,23^ and building in the necessary hardware required to have the robot meet the natural sea turtle locomotion while attempting to obtain the original form as closely as possible. To simplify the manufacturing process, the flipper geometry was simplified compared to a real turtle by removing the trailing edge serrations, scales and leading edge claw (Fig. S2a). However, the flipper cross-section remained as a turtle-inspired cambered hydro-foil profile^21,33^. Additionally, the locomotor pattern was minimised to three degrees of freedom. The locomotor patterns (Fig. 3a and Movie S1) were based on recent work by van der Geest et al.^5^, with the flipper equations of motion (plotted in Fig. 3b) in roll and yaw, expressed using a Fourier series with $n=8$, (Eqs. 1, 2) and pitch (flipper twist) expressed as a linear piecewise function (Eq. 3) with ($t=0$) set at the end of the sweep stroke (SS) and beginning of Recovery stroke one (RS1):

1 $${\theta }_{roll}\left(t\right) = {a}_{r}+{\sum }_{i=1}^{n} {a}_{ir}\,\mathit{cos}\left(i{w}_{r}t\right)+ {b}_{ir}\,\mathit{sin}\left(i{w}_{r}t\right)$$

2 $${\theta }_{yaw}\left(t\right) = {a}_{y}+{\sum }_{i=1}^{n} {a}_{iy}\,\mathit{cos}\left(i{w}_{y}t\right)+ {b}_{iy}\,\mathit{sin}\left(i{w}_{y}t\right)$$

3 $${\theta }_{Pitch}\left(t\right)=\left\{\begin{array}{c}{a}_{p1}t, 0\le t<{t}_{1}\\ {a}_{p2},{ t}_{1}\le t<{t}_{2}\\ {a}_{p3}t+{b}_{p3}, {t}_{2}\le t<{t}_{3}\\ {a}_{p4}t+{b}_{p4}, {t}_{3}\le t<{t}_{4}\\ {a}_{p5}, {t}_{4}\le t<{t}_{5}\\ {a}_{p6}t+{b}_{p6}, {t}_{5}\le t<{t}_{6}\end{array}\right.$$

Rotation axes were placed, considering the location of the turtle's shoulder and elbow joints based on video footage of real animals swimming in Australia's Great Barrier Reef (Fig. 3c). It is worth highlighting that despite the limb degrees of freedom being constrained to three, the robot could still perform the general patterns described by van der Geest et al.^5^.

Due to the turtle body's complex surfaces, additive manufacturing was extensively used throughout the assembly, with carbon fibre and aluminium only used on highly stressed components such as drive shafts and couples. The turtle was manufactured to a straight carapace length (SCL) of 610 mm from nine bonded sections to form the final geometry (Fig. S1b). Additionally, the turtle's main body was designed to fill with water to help neutralise buoyant and gravity forces. All rotation axes were supported with zirconia oxide bearings to remove unwanted wobble to the robotic limb and provide a smooth, corrosion-resistant rotation. Due to the additive manufacturing process, all cable management was done through specially printed internal channels within the turtle limbs to hide and protect the cables while allowing the necessary degree of freedom during operation. The robot's rear flippers were left static and thus uncontrolled. This was because when entering their general swimming routine, the rear flippers are almost motionless, tucked in, and pointing backwards to lower their parasitic drag^5,23^ (Movie S4).

### Soft robotic flipper design

The natural twisting of the sea turtle's forearm can be illustrated with a linearly varying twisting motion from the turtle's flipper tip to the elbow^5^. The exact level of twist along the flipper span can be found with $\theta \left(x\right)= \frac{{\vartheta }_{pitch}(t)}{s}x$. Where ${\theta }_{Pitch}\left(t\right)$ is the maximum amount of twist applied by the turtle at any point in time and $x$ the position of interest along the flipper span $(s)$.

To physically achieve a linear twist relationship, it is understood that when torque is applied to a cylinder of constant stiffness and diameter from a solid mechanics principle, a linear twist is produced down the cylinder length. Applying such a simple principle to achieve linear twist to a soft robotic flipper of varying cross-sections required the device to be manufactured in sections of varying stiffness.

**Figure 4:**
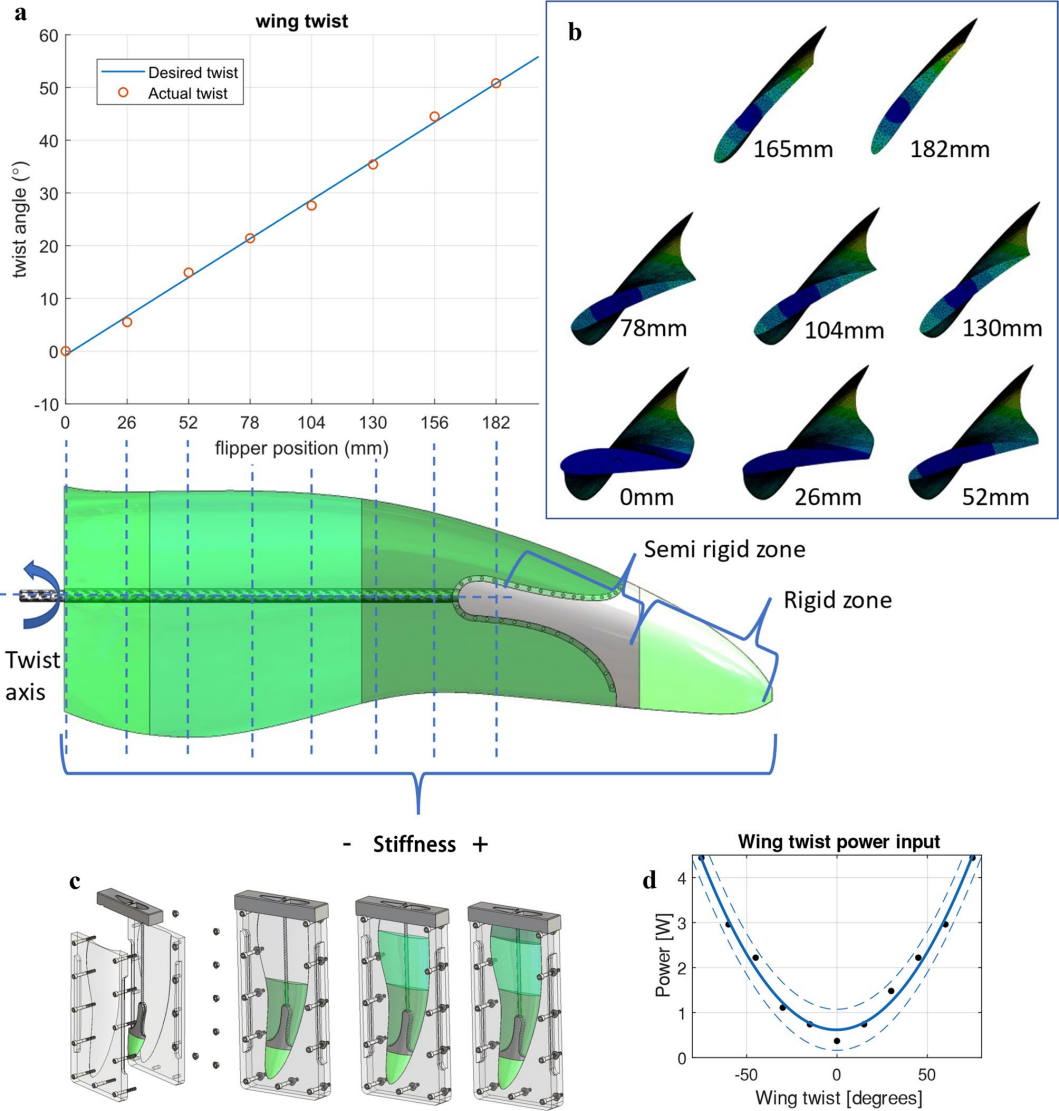
Twisting flipper design. (**a**) The plot of desired flipper twist against the actual flipper twist. The actual twist value position is projected down onto the CAD model's plan view, which also shows the twist axis's location and level of material stiffness throughout the flipper span (**b**) Cross sections taken from the FEA simulations that directly correspond to positions in Fig. 4a that show the level of flipper twist (**c**) CAD model showing split mould assembly with each of the three casting stages. (**d**) Power consumption to hold the flipper at each degree of twist from − 80° to + 80°.

The design process to achieve this took an iterative design approach by solving simulations within FEA of various composite flipper models (see “methods” section for FEA setup). This process involved slicing the flipper model into various sections and applying a variety of material properties to each section until a linear twist throughout the flipper span was achieved (Fig. 4a,b and Fig. S2b).

The optimal design was composed of a rigid carbon fibre spar that transmitted torque to a rigid flipper tip through a single actuator. As the actuated flipper tip rotates, this causes the flipper body to produce the required twist. The main body of the flipper was manufactured in three sections composed of a cast polyurethane rubber of stiffness shore A harness A40, A60 and A70 (Fig. 4c), with the softest material set at the flipper root and with the stiffest close to the flipper tip. The flipper tip was manufactured with a porous structure to allow the polyurethane to bond firmly to the rigid structure. This bond area produces a semi-rigid zone between the flipper tip and the main flipper body, where the polyurethane overlaps the rigid zone.

Manufacturing the flippers involved casting each layer simultaneously from the hardest to softest material, allowing the polyurethane to bond as though one single material at each interface. The mould was of a split design incorporating shrinkage risers and a holding jig for the carbon fibre spar and flipper tip in a simple single-part design (Fig. 4c and Fig. S3a).

Overall the design ensured the flipper could sustain large flexural loads while achieving the desired soft twisting actuation from $\pm$ 80° (Movie S2) while only consuming a maximum power input of 4.5 watts per flipper at 80° of twist (Fig. 4d). To understand the mechanical properties of the soft robotic flipper and evaluate its ability to support flexural loading, fatigue and single-point bending tests were performed on two identical flipper assemblies. During the single-point bending test, the flipper sustained a 14.8 Nm bending moment deflecting down at the wing tip 70 mm before the travel in the holding tool reached its max permissible deflection (Movie S3). The flipper may have resisted higher bending moment had the tool been designed to hold the wing higher from the machine bed. Fatigue tests were performed in a custom-built acrylic tank to actuate the flipper twisting motion for one complete cycle of − 73° to 35° (108° total) underwater (Movie S3 and Fig. S4a–d). The flipper sustained 922,154 cycles, equivalent to swimming 2395 km (refer to the “methods” section for additional details). At the end of fatigue testing, the leading cause of failure was the seizing of the servo transmission. The flipper showed no evidence of failure at the casting interfaces however, at the flipper trailing edge, the adhesive had begun to separate from the wing root (Fig. S4e).

### Robot testing overview

All swim tests conducted measured power consumption, swim speed, thrust and lift generation with the flapping frequency held constant at 0.23 Hz based on freely swimming wild green sea turtles^5,22^. A total of three different testing procedures were followed, firstly constraining the robot, so it could not move. This gave insight into the turtle's propulsion from a standstill and additionally served as a benchmark for dynamic swimming tests.

The second test allowed the robot to freely swim however, the robot had to overcome a constant 2.5 N dynamic friction load within the system generated by the linear rail. This test allowed the restricted swim speed to be gathered based on the robot overcoming the friction loads and was used as a reference for calculating the possible swim speeds without friction.

Finally, the robot was towed up to speed before it could take over to propel itself. During tow testing, the robot would be towed up to 0.6 m/s based on the cruising speeds of wild sea turtles^23–25^ until the robot began the downstroke, from where it would begin to accelerate on its own (Fig. S6b). With testing results showing propulsion was only produced during DS and SS, the loss in swim speed (without friction forces) during the remaining swim cycle was derived from Newtonian physics by integrating the acceleration term with respect to time and substituting in the hydrodynamic force with:

$$\Delta {v}_{2}=\frac{\rho {A}_{p}{{c}_{d}\left({v}_{o}-\Delta {v}_{1}\right)}^{2}\Delta t}{2m}$$

where $$m$$ is the mass of the robot, $$\rho$$ the fluid density, $${A}_{p}$$ is the projected frontal area of the robot and $$({c}_{d})$$ the robot drag coefficient. This calculation was iterated with a constant time step $$(\Delta t)$$, with the initial swim speed ($${v}_{0})$$ defined as the maximum velocity obtained during the swim test with $$\Delta {v}_{1}=0$$ for the first iteration. For the following iterations $${v}_{0}$$ was calculated as

$${v}_{0}={v}_{0}-\Delta {v}_{2}$$

This process was iterated to cover the time when no thrust was produced. The complete swim speed cycle was created for a freely swimming sea turtle by compiling all the velocity data from tow testing (Fig. S6b) with the loss in swim speed calculations. From this point, the swim speed was differentiated with respect to time and compared back to the original thrust values (Fig. S6a) to double check and verify that the swimming speed was possible based on the measured thrust values.

**Figure 5:**
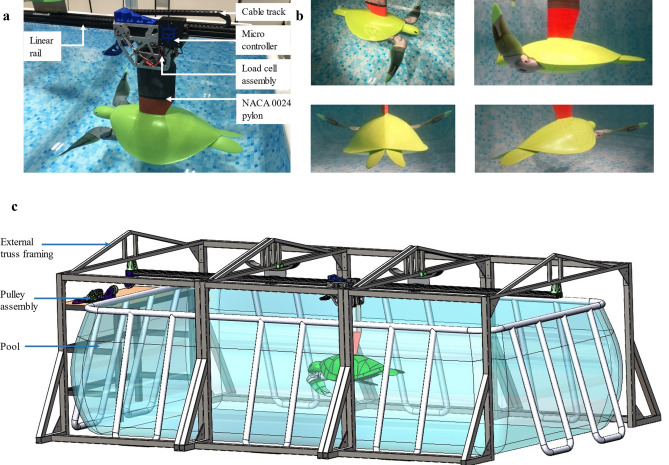
Robot and test rig assembly. (**a**) Robot, pylon and load cell assembly. (**b**) Footage of robot swimming from various view angles see Movie S4. (**c**) Complete CAD model of test rig assembly showing external truss framing to support linear rail above the pool.

To remove the need for a complex control system and make force data and power consumption easily obtainable, the robot was attached to a pylon comprised of a NACA 0024 cross-section with a varying chord. The pylon was bolted to a 40 kg load cell for measuring lift forces and connected to a linear rail and cable track (Fig. 5a,b). Overall, the robot and pylon assembly weighed 30 kg when the cavities within the robot filled with water.

Drag and constrained thrust forces were measured from the pully assembly with a built-in 20 kg load cell (Fig. 5c and Fig. S5a–c). Thrust forces during free swimming were calculated based on the robot's acceleration ($\dot{v}$), friction load (${F}_{f}$) and hydrodynamic drag force with:

$${F}_{T}=m\dot{v}+ \frac{\rho {A}_{p}{c}_{d}{\left(v\right)}^{2}}{2}+{F}_{f}$$

where $m$ is the mass of the robot, $\rho$ the fluid density, ${A}_{p}$ is the projected frontal area of the robot, ${c}_{d}$ the robot drag coefficient, $v$ the robot velocity and ${F}_{f}$ the linear guide system friction. The robot's drag coefficient was found using both CFD with verification from tow testing by subtracting the 2.5 N dynamic friction load within the system generated by the linear rail assembly during simple tow tests. This process involved towing the robot through the pool with the limbs held stationary (Figs. S6c–e and S9).

The linear rail assembly was bolted to an aluminium extrusion truss frame that saddled a freshwater pool of dimensions 4 m long, 2 m wide, and 1 m deep and gave a rigid foundation to the overall system, and positioned the robot centrally in the pool as shown in Fig. 5 and Fig. S5d. The depth of the pool used was deemed as suitable as green sea turtles spend most of their time in the shallow fringing reefs^5^ as seen in Movie S4.

### Robot testing power and force

**Figure 6:**
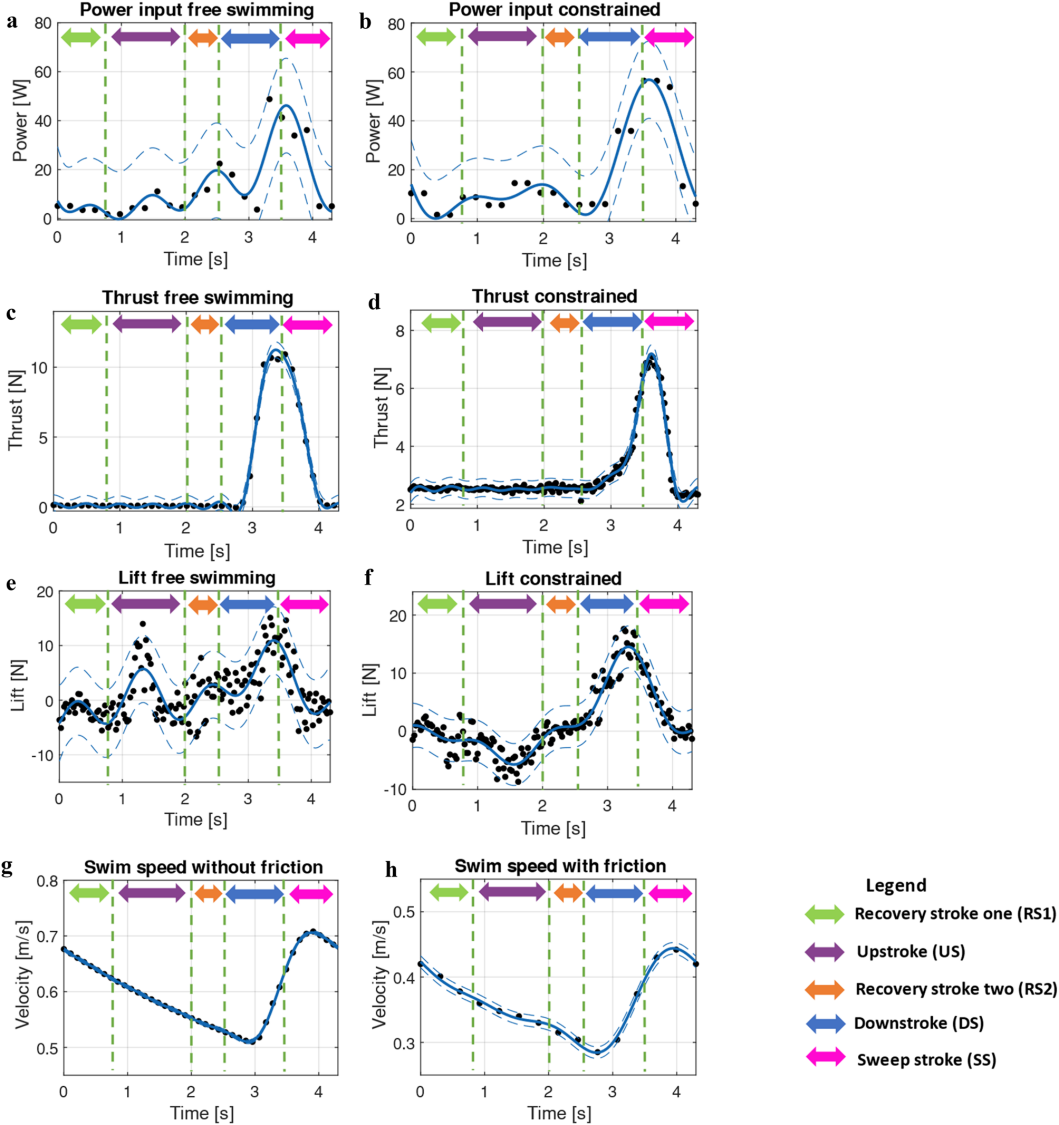
Testing data and results, green arrow showing the amount of time spent during RS1, purple arrow showing the amount of time spent during the US, orange arrow showing the amount of time spent during RS2, blue arrow showing the amount of time spent during the DS and magenta arrow showing the amount of time spent during SS. (**a**) Power consumption against time with pulley assistance to bring robot up to speed (**b**) Power consumption against time for the constrained operation. (**c**) Thrust generation for the freely swimming robot. (**d**) Recorded load cell thrust generation for constrained operation. The plot includes a 2.5 N vertical offset to account for the constant dynamic friction load. (**e**) Recorded load cell data plotted against time for the lift force with pulley assistance. (**f**) Recorded load cell data plotted against time for constrained operation. (**g**) Predicted swim speed for the robot without any friction from the test rig assembly. (**h**) Swim speed data for the robot without pully assistance, meaning the robot had to overcome a constant 2.5 N system friction load.

This section will continuously refer to Fig. 6, where the various testing results of power consumption, thrust, lift and swim speed are detailed. All power and force plots represent constrained operation and testing with the pulley system to get the turtle up to its cruising speed. The vertical green dashed lines represent the location in time for each of the turtle's five swim stages as outlined in Fig. 3a.

Referring to Fig. 6a,b, the power consumption for both constrained and free swimming (towed up to speed) can be observed. Our findings show that the power consumption during RS1, US, and RS2 always lay below 20 watts, with the only significant energy consumption occurring during DS and SS. Additionally, the unconstrained swimming robot demonstrated a lower energy consumption, along with higher thrust values and lower lift values (Fig. 6a–f). This finding was expected as the swimming pattern is extracted from freely swimming wild green sea turtles, as demonstrated by van der Geest et al.^5^. The noticeable decrease in thrust and increase in the lift for the constrained robot is likely due to adverse pressure gradients causing excessive flow separation, making the downstroke less efficient, with the sweep stroke not being largely affected due to the relative velocity difference.

For free and constrained operation, no evidence of thrust production was detectible during the upstroke (Fig. 6c,d). However, a positive lift was generated for freely swimming, with a negative lift generated during constrained operation. This finding supports the work of van der Geest et al.^23^, where green sea turtles are shown to produce a drag-reducing upstroke that utilises lift forces on the turtle's flippers to help bring the flippers up, potentially lowering energy consumption. Additionally, a non-propulsive upstroke was also discovered by David T. Booth^34^ for green sea turtle hatchlings.

### Robot testing swim speeds

Referring to Fig. 6g,h, the robot swim velocities are detailed for one complete wing oscillation. The velocity data in Fig. 6h represents the exact swim velocity obtained by the robot without any pulley assistance thus, the robot had to overcome the constant 2.5 N friction force within the linear rail assembly. A peak swim speed of 0.45 m/s was obtained, producing an average swim speed of 0.361 m/s. As seen in Fig. 6g the swim speed for a freely swimming turtle without any friction is displayed. A peak swim speed of 0.71 m/s was obtained, with an average swim speed of 0.602 m/s, closely matching literature for freely swimming sea turtles^23–25,35^. Based on the average swim speed, the Reynolds number was calculated based on the straight carapace length ($${D}_{SCL})$$ applying $$Re= \frac{v\rho {D}_{scl}}{\mu },$$ to obtain values of 220,000 and 367,000. Where $$v$$ is the average swim speed $$\rho$$ the fluid density and $$\mu$$ the fluids dynamic viscosity. At the same average swim speeds, the Strouhal number (St) of 0.24 and 0.4 was calculated by applying, $$St=\frac{Df}{v}$$ where $$D$$ is the peak-to-peak distance of wing oscillations $$f$$ the flapping frequency and $$v$$ the average swim velocity. The values of St found during testing lie in the region observed in other flying and swimming animals of 0.2–0.4^36–39^.

## Discussion

In this work, we disclose a new soft robotic sea turtle that can closely bio-mimic the natural sea turtle's general swimming locomotor patterns as described by van der Geest et al.^5^. Developing the robot meant overcoming two significant obstacles. Firstly, designing the mechanical system to accomplish the locomotor pattern, and secondly, keeping the natural sea turtle form. The essential contribution to achieving this was our new soft twisting flipper that can replicate the natural animal's morphing ability while still being capable of supporting large flexural loads. To the best of our knowledge, this makes our robot the first attempt at producing a robotic sea turtle that can produce the general swimming patterns of a wild animal.

This work's primary purpose was to outline the design of the biomimetic robot to help better understand the sea turtle's propulsion mechanism. Gaining this understanding through conventional animal testing would have been highly challenging in our current paradigm of ethical approvals, especially when endangered animals are concerned. However, although we reverse engineer the sea turtle to take a robotic form, we demonstrate that the robot can swim at 0.6 m/s with a flapping frequency of 0.23 Hz. This alone is a notable finding as previous studies on wild green sea turtles demonstrate that a swim speed of 0.6 m/s is obtained at a flapping frequency of around 0.23 Hz^22^. Additionally, in our own research expedition in Australia's Great Barrier Reef^5^, we also found that green sea turtles produced an average limb beat cycle of 0.234 Hz, and it is understood that the average swim speed of sea turtles is 0.6 m/s^23–25^. We use these findings of flapping frequency and swim speed as a form of validation for the suitability of our robot as a tool for understanding sea turtle propulsion methods. Additionally, at this flapping frequency and swim speed, a Strouhal number of 0.24 is obtained, which directly lies in the region observed in other swimming and flying animals and is understood to contribute to their efficient mode of transport^36–39^.

Our robot also demonstrated that during the animal's regular swimming routine, propulsion is only generated for 30% of the overall cycle, with the animal entering an energy-saving glide during the remaining 70%. This is made evident when observing the power and thrust curves, as seen in Fig. 6, with the only substantial power consumption occurring during the DS and SS, along with the thrust forces only being registered during the same period in time. During the upstroke, a positive lift force was generated, suggesting sea turtles take advantage of their flippers' hydrodynamic loading to help bring them up to the top of the stroke. This may support the findings of van der Geest et al.^23^ that sea turtles produce a passive drag-reducing upstroke for conserving energy by allowing the flippers to enter a near-equilibrium state that prevents cross flows due to pressure differentials^23^.

Some caveats require discussion with our findings due to the simplification of various systems, equipment and methodology. Firstly having the robot constrained in the vertical axis so lift forces could be obtained prevents the turtle from pitching up and down due to the vertical forces during swimming. The removal of the pitching motion could cause the robot to swim slightly quicker due to the body keeping a constant angle of attack to the oncoming flow. Additionally, all tests were performed and calculations made with freshwater, not typical seawater that the animal would swim in. Though both these simplifications likely affect the swim speed, they were both essential for the success of the experiments as the lift forces would have been difficult to obtain without the vertical constraint, and the amount of salt required to create the correct saltwater density was extreme at approximately 200 kg. Additionally, constraining the robot meant advanced control systems were not needed, thus allowing for a more detailed analysis of what forces are generated during the regular swimming routine. Another talking point to simplifications can be observed in the pool size. As discussed earlier in the manuscript, the depth of the pool is deemed appropriate as sea turtles spend most of their time in the fringing reefs^5^, often only 1 m in depth. However, the pool length of only 4 m which resulted in limited flipper oscillations before the robot ran out of runway. This issue could be remedied in future work with the robot swimming autonomously in a large pool or even in an ocean environment.

Obviously, this work primarily takes geometric and kinematic data from video footage^5,23^ rather than conventional methods that typically use a live or dead specimen. This method of data collection could potentially lead to discrepancies geometrically and kinematically. However, it must also be noted that all sea turtles vary in shape, size, mass, and flipper motion, so although video footage and photos are used when comparing the motion of our robot with natural sea turtles, there is a minimal argument that can be made regarding its ability to mimic its biological counterpart.

Also worth noting is that the turtle geometry used in this study is based on our previous work^23^, where we detail the development of a turtle geometry that we compare with biological data, including the drag coefficient of the carapace (without flippers)^40^, frontal area (including flippers), mass (including flippers), and SCL^41^ to achieve valuable correlations and give confidence in our turtle geometry.

By removing features such as leading-edge claws and trailing-edge serrations of our turtle model could lead to hydrodynamic discrepancies from our model to its biological counterpart. However, this was, unfortunately, a necessary detail to achieve the extremely tight budget and immensely helped simplify the manufacturing process by reducing the need for complex machining of the split mould assembly, as seen in Fig. S3.

Though several caveats are evident in our work, we believe we have created what is the best understanding of the sea turtle's propulsive routine during its regular swimming pattern. Additionally, we have accomplished this without any animal testing by designing what we believe is the world's first and only robotic sea turtle that was solely developed to help understand the sea turtle's propulsion methods. We hope this work will help inspire others to develop robots as a substitute for their biological counterpart.

In future work, we plan to use this robot to identify what unique flow features, including wall effects, are responsible for the thrust generation during the DS and SS, along with how this could be optimised and developed for the next generation of robotic ocean exploration devices.

## Materials and methods

### Design and fabrication of sea turtle chassis

The foundation of the robot design is based on works by van der Geest et al.^5,23^, applying both the swimming locomotor patterns^5^ and the green sea turtle CAD model^23^ to help set the design foundation. Van der Geest et al. describe the turtle's locomotor patterns of the flipper tip, flipper elbow and flipper twist using a Fourier series as:

$$x\left(t\right)= {s}_{f}({a}_{x}+{\sum }_{i=1}^{n} {a}_{ix}\,\mathit{cos}\left(i{w}_{x}t\right)+ {b}_{ix}\,\mathit{sin}\left(i{w}_{x}t\right))$$

$$y\left(t\right)= {s}_{f}({a}_{y}+{\sum }_{i=1}^{n} {a}_{iy}\,\mathit{cos}\left(i{w}_{y}t\right)+ {b}_{iy}\,\mathit{sin}\left(i{w}_{y}t\right))$$

$$z\left(t\right)= {s}_{f}({a}_{z}+{\sum }_{i=1}^{n} {a}_{iz}\,\mathit{cos}\left(i{w}_{z}t\right)+ {b}_{iz}\,\mathit{sin}\left(i{w}_{z}t\right))$$

$$\theta \left(t\right)=\left\{\begin{array}{c}-{\theta }_{ds}, \quad0\le t<{t}_{1}\\ {\sum }_{n=i}^{n}{a}_{dsi}\mathit{sin}({b}_{dsi}t+{c}_{dsi}),\quad {t}_{1}\le t<{t}_{2}\\ {\theta }_{us},\quad {t}_{2}\le t<{t}_{3}\\ {\sum }_{n=i}^{n}{a}_{usi}\mathit{sin}({b}_{usi}t+{c}_{usi}),\quad {t}_{3}\le t<{t}_{4}\end{array}\right.$$

where the position of the flipper tip and elbow can be expressed with $x\left(t\right), y(t)$ and $z(t)$ and flipper twist expressed with $\theta (t)$. The variable ${s}_{f}$ is used to scale the equations to suit any sized turtle based on a reference straight carapace length of 610 mm^5^.

The green sea turtle CAD model from van der Geest et al. was inserted into a CAD (SolidWorks, Waltham, Massachusetts, USA) assembly along with the three-dimensional data points of the locomotor pattern. This was achieved by solving the equations within CAD using the "Equation Driven Curve" function to create a part consisting of only three-dimensional data points representing the turtle's locomotor pattern. Having the turtle CAD model and locomotor patterns within a CAD assembly allowed for an iterative design approach to then take place for developing the robotic limb. Once the final design was complete (Fig. S1a), a virtual clone within CAD (Movie S1) could be used to verify the locomotor patterns.

The robot's chassis was additively manufactured from PLA + (Esun, Shenzhen, China) filament printed on an FDM 3D printer (Caribou MK3s, Remagen, Germany). As shown in Fig. S1b–f the chassis was manufactured in twelve sections (Fig. S1b), with each part sliced (Simplify3D version 4.1, Cincinnati, Ohio, USA) with a 0.4 mm nozzle at 0.2 mm layer heights, 12 top and bottom layers, 6 walls, and 40% honeycomb infill. The chassis was then bonded together with methylene chloride before receiving an automotive quality paint job (Fig. S1c,d). All surface fasteners were recessed or countersunk to help lower parasitic drag. Three access hatch locations were placed under the chassis to access roll motors and electrical connections (Fig. S1e). The elbow and shoulder joints were also additively manufactured however were left as printed without paint (Fig. S1f).

### Design, fabrication, FEA setup and testing of soft robotic flipper

As seen in Fig. S2, the design process to achieve the soft robotic flipper took an iterative design approach using commercial FEA software (ANSYS 2021 R2, Pennsylvania, USA). Simulations were set up to test various material layups and properties to inspect their effect on twisting motion. The first design iteration began with designing a layup that could potentially work using our mechanical design intuition coupled with the solid mechanic's principle of torsion previously mentioned in the main manuscript. From this point, the FEA results could be reviewed to see what potential layup could be applied to improve the results. This process was followed until the desired twist profile was achieved. Simulation boundary conditions consisted of a fixed support at the wing root with bonded sections between each material layup. To create the twisting motion, a frictionless contact was applied to the spar and flipper interface, with a remote angular displacement applied to the carbon fibre spar. Material properties were simplified by estimating the Young's modulus based on the shore A hardness using:

$${log}_{10}E=0.0235{S}_{A}-0.6403$$

where $E$ is the elastic modulus in MPa and ${S}_{A}$ the shore A hardness. The carbon fibre spar had mechanical properties of ultimate bending stress ($\sigma =822\text{ MPa}$) and Young's modulus of $(E=85\text{ GPa})$. Mechanical properties were found through 3-point bending tests (H10KS Universal testing machine, Tinius Olsen Ltd., Surrey, England).

The final design used a solid 5 mm diameter carbon fibre spar that was lubricated just before the casting process to ensure smooth rotation between the flipper and spar interface. The flipper tip and spar were assembled into the mould assembly before casting, as seen in Fig. S3a. Each cast section was from shore A hardness polyurethane rubber (F140, F160, F170 Barnes, Moorebank, NSW, Australia). The 40 shore A section was approximately 42 mm long at the flipper root, with a 60 shore A section of 121 mm and finally the 70 shore A section of 71 mm (Fig. S3b). Each section was directly cast one after the other to create a single casting with excellent bond strength between each material (Fig. S3c).

Fatigue testing was performed with a custom-manufactured water tank made from 18 mm thick clear acrylic that would actuate the flipper through an Arduino Nano controlled (Arduino.cc, Somerville, MA, USA) servo motor (Fig. S4). The system was designed to pause the actuation if a drop or rise in current over the regular operation was detected, then save the cycle count to an SD card.

The flipper bending test was performed with a custom-manufactured holding jig, as shown in Movie S3 that was installed into a universal testing machine (H10KS, Tinius Olsen Ltd., Surrey, England). The test was performed at 1 mm/min until max permissible deflection was achieved due to design constraints.

### Force and swim speed measurements

Swim speeds were found by measuring the time taken for the robot to pass markers of width 17.5 mm spaced apart a distance of 30 mm along the linear rail assembly (Fig. S5a). Time measurements to pass the markers were obtained by filming the robot with a camera (GoPro Hero 10, GoPro, San Mateo, California, USA) at 240 frames per second and 2 K resolution. The camera was towed along with the robot down a separate linear rail that ran parallel to the robot's swim direction (Figs. S5a and S6b).

For constrained operation, thrust forces were obtained by installing an additional pulley, as seen in Fig. S5b, to allow the robot to swim against the pully load cell (Fig. S5b,c). As this setup only allowed load cell thrust measurements to be obtained while constrained, thrust for swimming operation was obtained based on the robot's acceleration, drag force and friction within the linear rail assembly as described in the main manuscript. The friction forces caused by the linear rail assembly were obtained by operating the equipment without the robot. The dynamic friction load within the system was 2.5 N (Fig. S6c,d) regardless of operating speed between 0.3 and 0.6 m/s.

### Electrical system robot

The simplified schematic in Fig. S7 shows the electronic system used for the control of the turtle flippers. This required a suitable power supply for the six Savӧx SW1210SG servos, (SAVOX, Taichung City, Taiwan) a microcontroller (MCU) (Particle Industries Inc., San Francisco, USA) with enough timer resources to produce the PWM signals for the control of the six servos, and to generate the real time interrupts (RTI) to update the servo set positions so the flipper can follow the desired trajectory accurately. The hardware also needed to take external input to control the actions of the system for the various tasks such as starting/stopping the turtle swimming or changing the flapping frequency.

The Particle Photon MCU was chosen as it had sufficient timer resources capable of 7 independent PWM signals and a built-in WIFI module to facilitate wireless control and over-the-air (OTA) firmware updates. The main power supply was a variable 15A DC supply set to the servos recommended operating voltage of 7.4 V. This power supply was external from the gantry allowing the operator of the robot to disable the supply in the event an unexpected system failure occurred. Two more on/off switches were used to separate the MCU and servos power supply control, allowing for the MCU firmware to be updated and tested without the servos operating. A buck converter was used to step down the main 7.4 V supply to the 5 V required by the MCU. Brownout protection was implemented to prevent voltage drop in the main power lines due to possible large changes in the servos required torque moving between set positions.

### Software

The firmware for the controller was written in C++ using Particles Web IDE. The software was split into two parts: one the control of the servos for the flapping motion and moving between desired poses and two the wireless operator controls. For the control of the servos a RTI and a look-up table (LUT) containing the set positions for each step in the flapping pattern are used to control the flippers. Using Eqs. (1), (2), (3) the LUT is generated for a 50 ms interval across the 4.3 s period of the motion, resulting in 86 steps. The 50 ms time interval can be varied by the operator to alter the robot's flapping frequency. The firmware uses the angular set positions in the LUT to calculate the required duty cycle on period of the PWM signal 800–2200 us for ± 80 degrees range of motion. To ensure the flipper will closely follow the desired trajectory and smooth motion, each step in the pattern is interpolated between taking 3 sub steps to move between steps. This means the RTI used to update the servo set positions will occur at the rate described by ${T}_{interrupt}=\frac{{T}_{step}}{n}$ where ${T}_{step}$ is the period of one step, ${T}_{interrupt}$ is the RTI period and $n$ is the number of interpolation steps.

For moving the robot's flippers between desired poses such as the zero position or start position of the swimming pattern, interpolation is again used. The current set position of the flippers is always stored to ensure it can be used as the starting point of the interpolation to the new pose when a command to move is received, this prevents the servos from jumping between positions. The number of steps between the two set positions and the time interval between the interpolation steps can be specified in the firmware to allow smooth control of the flippers. To prevent collisions between the flippers and the body, a set of pre-planned poses are used with the flipper's zero position always used as the first position because from any position, the flippers can reach the zero position without the possibility of collision.

The operator control of the system is facilitated by a standard state machine approach, using states such as swimming, stopped, reset, move to zero position, move to low drag position etc. The states are selected by the operator using a web-based GUI application written in HTML that communicates to the Photon MCU over WIFI using the Particle API functions.

### Electrical system testing rig

The testing rig electrical system (Fig. S8a) is composed of a graphical user interface (GUI) programmed on LabView (National Instruments, Austin, Texas, USA), two electric systems (one to control the motor and another for the data acquisition of the load cells), the pulley system and the load cell assemblies (lift and drag).

The GUI (Fig. S8c) was able to turn on and off the motor of the pulley system in a synchronous way with the load cell data acquisition system using serial communication between the computer and the microcontrollers. Furthermore, when the program starts, it records the data from the load cells into a txt file. The information on the txt file contains the data of the reading sample, the information coming from both load cells. When the system stops, it saves the file into a selected folder.

The motor control system (Fig. S8b) consisted of an electric circuit containing an A4988 stepper motor driver (Allegro Microsystems, Manchester, New Hampshire, USA), a microcontroller Arduino NANO and a variable DC power supply skyRC efuel 540w (SkyRC Technology, Guanlan, Shenzhen, China). The Arduino controlled the angular speed of the motor in revolutions per minute (RPM) using the A4988 driver with a micro step of a 1/4 step to ensure smooth motor steps during the low initial speeds. Furthermore, to avoid high acceleration loads, linear velocity was defined as: $v=0.2t$. Where "v" is linear velocity and "t" is time. Moreover, a pulley (r = 0.0225 m) attached to a stepper motor, "NEMA 17", was used to transform the angular speed to a linear speed. Then the linear speed could be calculated as: $v=\frac{2\pi r(RPM)}{60}$.

Finally, the data acquisition system (Fig. S8b) consisted of a second Arduino NANO connected to two SparkFun load cell amplifiers, HX711 (SparkFun Electronics, Niwot, Colorado, USA). The HX711 were connected to two load cell assemblies, one for the lift and one for the drag. To record the lift, a 20 kg load cell (YZC-1B, XIN NUO QI, China) was used, while a load cell of 40 kg (YZC-1, XIN NUO QI, China) was used for the drag force. The sampling frequency of the system was 40 Hz.

### CFD analysis and turtle geometry

In our previous work^23^, we detail the development of a turtle geometry that we compare with biological data, including the drag coefficient of the carapace (without flippers)^40^, frontal area (including flippers), mass (including flippers), and SCL^41^ to achieve valuable correlations and give confidence in our turtle geometry. This geometry was used to develop our robotic sea turtle as described in the main manuscript. We use CFD as a tool to help evaluate the drag coefficient against the original more organic geometry to act as a form of validation. We found our robot produced a drag coefficient of 0.16 when the flippers/wings were held stationary, as per Fig. S9, aligning well with the values obtained in our previous work of 0.16, also obtained from CFD with the results available in the supplementary folder of that work^23^. This gave us confidence that the robot's geometry was as close as physically possible to the original geometry, given the challenging nature of creating the limbs as previously described. The simulation was set up to solve with the $k\omega SST$ model using commercial CFD software ANSYS Fluent (ANSYS 2021 R2, Pennsylvania, USA) with all model parameters left in their default settings. The Fluent polyhex core mesh was used, consisting of 81,001,792 nodes and 38,886,857 elements. The mesh was refined down to a ${y}^{+}$ of 0.75 to accurately solve the flow down to the viscous sublayer. The computational domain was modelled as per the swimming pool used and halved at the symmetry plane with the symmetry plane boundary condition applied. The pool surface of the computational domain was set as a free slip wall, with the pool walls and floor set as zero slip moving walls at the inlet velocity of 0.6 m/s. The turtle geometry was set as a smooth no-slip wall condition. Convergence reports were set up to study the standard deviation of the velocity magnitude at the outlet, the area-weighted average of the wall ${y}^{+}$, the lift, drag and scaled residuals.

## Supplementary Information

Supplementary MATLAB Code.

Supplementary Movie 1.

Supplementary Movie 2.

Supplementary Movie  3.

Supplementary Movie 4.

Supplementary Figures.

Supplementary Information.

## Data Availability

All data sets for the current study are available from the authors on reasonable request via contacting the corresponding author.
