# Supplementary material for: Soft-robotic green sea turtle (Chelonia mydas) developed to replace animal experimentation provides new insight into their propulsive strategies
Source: Sci Rep. 2023 Jul 25;13:11983. doi: 10.1038/s41598-023-37904-5 (PMC10368674; doi:10.1038/s41598-023-37904-5)
Supplement: Supplementary file 1 — Supplementary MATLAB Code. [file 41598_2023_37904_MOESM1_ESM.pdf]

```
%time only defined from 0 to 8.6 seconds. Function output is in degrees
interval=0.05;
t=0:interval:4.3-interval;
```

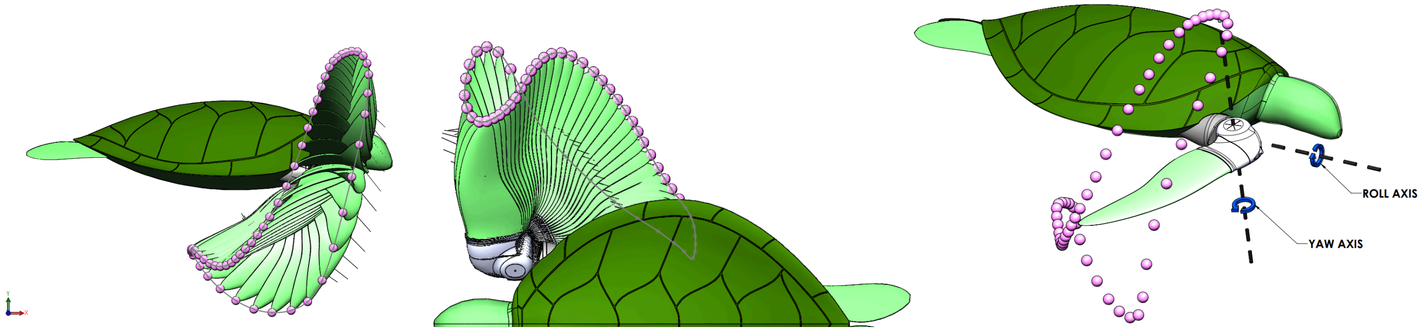


```
%contant values solved from non-linear least sqaures for roll axis
a0r = 3.278;
a1r = -60.12;
b1r = -19.3;
a2r = -5.566;
b2r = 15.64;
a3r = -0.08129;
b3r = 3.339;
a4r = 0.4491;
b4r = -0.3352;
a5r = -1.401;
b5r = -0.1525;
a6r = -1.423;
b6r = -0.6598;
a7r = -0.8562;
b7r = -0.2633;
a8r = -0.5337;
b8r = 0.09667;
wr = 1.461;

%function for roll axis
roll =  a0r + a1r*cos(t*wr) + b1r*sin(t*wr) + a2r*cos(2*t*wr) + b2r*sin(2*t*wr) +
a3r*cos(3*t*wr) + b3r*sin(3*t*wr) + a4r*cos(4*t*wr) + b4r*sin(4*t*wr) + a5r*cos(5*t*wr) +
b5r*sin(5*t*wr) + a6r*cos(6*t*wr) + b6r*sin(6*t*wr) + a7r*cos(7*t*wr) + b7r*sin(7*t*wr) +
a8r*cos(8*t*wr) + b8r*sin(8*t*wr);

%contant values solved from non-linear least sqaures for yaw axis
 a0y = 0.8961;
 a1y = -25.16;
 b1y = -22.35;
 a2y = -8.635;
 b2y = -2.142;
 a3y = -2.605;
 b3y = 0.2852;
 a4y = -0.9021;
 b4y = -0.8331;
 a5y = -0.1804;
 b5y = 0.4037;
 a6y = -0.01423;
```

```
 b6y = 0.1734;
 a7y = -0.06358;
 b7y = 0.1808;
 a8y = -0.0916;
 b8y = 0.1716;
 wy = 1.461;

%function for yaw axis
yaw =  a0y + a1y*cos(t*wy) + b1y*sin(t*wy) + a2y*cos(2*t*wy) + b2y*sin(2*t*wy) +
a3y*cos(3*t*wy) + b3y*sin(3*t*wy) + a4y*cos(4*t*wy) + b4y*sin(4*t*wy) + a5y*cos(5*t*wy) +
b5y*sin(5*t*wy) + a6y*cos(6*t*wy) + b6y*sin(6*t*wy) + a7y*cos(7*t*wy) + b7y*sin(7*t*wy) +
a8y*cos(8*t*wy) + b8y*sin(8*t*wy);

t1=0:interval:0.4;   % for a time of 0 to 0.398 seconds
t2=0.4+interval:interval:2.3;   % for a time of 0.398 to 2.309 seconds
t3=2.3+interval:interval:2.7;  % for a time of 2.309 to 2.707 seconds
t4=2.7+interval:interval:3;    % for a time of 2.707 to 3.026 seconds
t5=3+interval:interval:3.65;       % for a time of 3.026 to 3.663 seconds
t6=3.65+interval:interval:4.3-interval; % for a time of 3.663 to 4.3 seconds


%flipper twist/pitch piece wise function
theta1 = (17500/199)*t1; % for a time of 0 to 0.398 seconds
theta2 = ones(size(t2))*35; % for a time of 0.398 to 2.309 seconds
theta3 = -(17500/199)*t3 + (94745/398); % for a time of 2.309 to 2.707 seconds
theta4 = -225.705*t4 + 610.984; % for a time of 2.707 to 3.026 seconds
theta5 = ones(size(t5))*-72; % for a time of 3.026 to 3.663 seconds
theta6 = (72000/637)*t6 - (309600/637); % for a time of 3.663 to 4.3 seconds

[t1,t2,t3,t4,t5,t6];
pitch=[theta1,theta2,theta3,theta4,theta5,theta6];

%plot
plot([t,t+4.3],[roll,roll],'o','Color',[0.1, 0.7, 0.1],'LineWidth',1.1)
hold on
plot([t,t+4.3],[yaw,yaw],'o','Color',[0.5,0.1,1],'LineWidth',1.1)
plot([t,t+4.3],[pitch,pitch],'o','Color',[1,0.75,0.25],'LineWidth',1.1)
grid
title('Flipper motion @ 0.23Hz')
xlabel('time [s]')
ylabel('\theta [\circ]')
legend('roll','yaw','pitch')
xlim([0 4.3])
ylim([-80 80])
set(gca,'FontSize',12)
xticks(0:0.5:8.5)
yticks(-80:20:80)
hold off
```

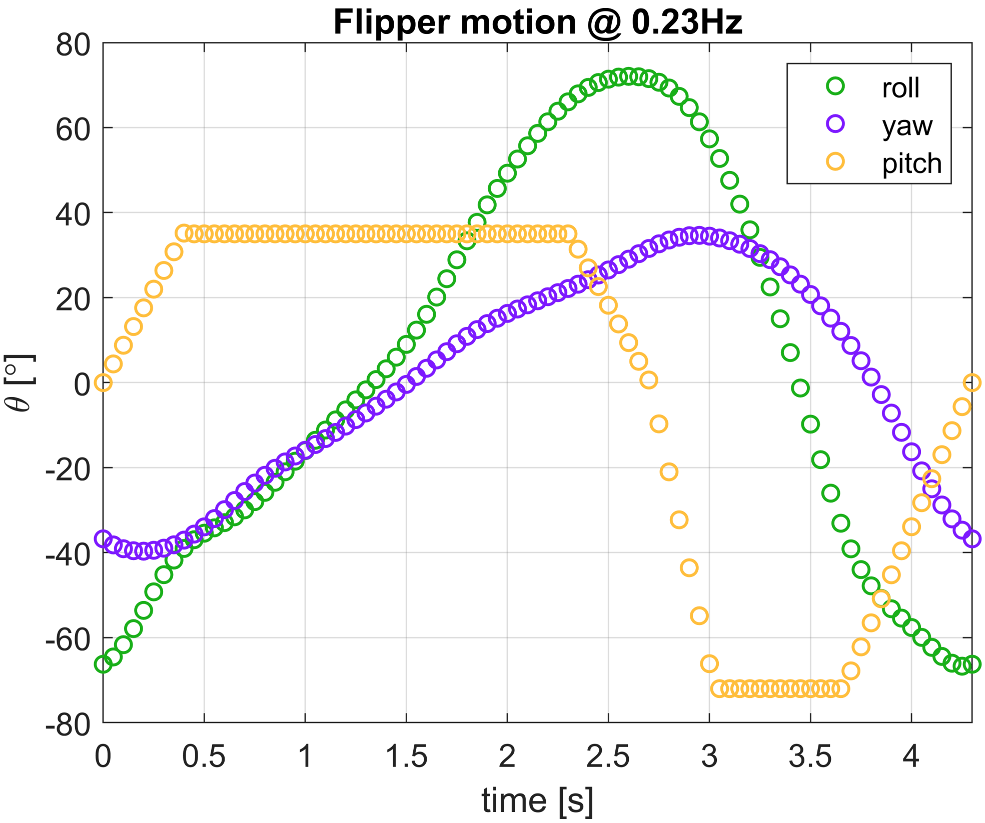

Flipper motion @ 0.23Hz
θ [°]
time [s]
roll
yaw
pitch
80
60
40
20
0
-20
-40
-60
-80
0
0.5
1
1.5
2
2.5
3
3.5
4
