# Supplementary material for: Soft-robotic green sea turtle (Chelonia mydas) developed to replace animal experimentation provides new insight into their propulsive strategies
Source: Sci Rep. 2023 Jul 25;13:11983. doi: 10.1038/s41598-023-37904-5 (PMC10368674; doi:10.1038/s41598-023-37904-5)
Supplement: Supplementary file 7 — Supplementary Information. [file 41598_2023_37904_MOESM7_ESM.pdf]

Soft-robotic Green sea turtle (*Chelonia mydas*) developed to replace animal experimentation provides new insight into their propulsive strategies.

Supplementary documents contain:

1- **Flipper_kinematics_robot_4_3_second_rotation.pdf**

This file provides a MATLAB program for solving the robots equations of motion for the Roll, Pitch and Yaw (twist) of the flipper.

2- **Supplementary figures.pdf**

This file provides all static supplementary figures S1 - S9

- **Fig. S1. Sea turtle robot chassis. (a)** Rendered CAD model of final robot design **(b)** 3D printed sections **(c)** Bonding of each section to form complete chassis. **(d)** Painting of chassis. **(e)** Access hatch locations to access electrical components. **(f)** Assembly of 3D printed limb onto chassis.
- **Fig. S2. Soft robotic flipper design. (a)** Three different green sea turtle flipper geometries showing leading edge claw and trailing edge serrations. It can be observed that each flipper has a slightly different shape. Additionally, the image on the right shows the final geometry and cross-section profile that was derived for the robot. **(b)** Design iterations starting from the top left image showing the first design iteration. The larger geometries shown below are the final iteration, with the left image showing the typical mesh used for FEA simulations.
- **Fig. S3. Flipper manufacturing. (a)** Split mould assembly showing flipper tip and carbon fibre spar installed ready for casting **(b)** Casting levels for each compound. **(c)** Pair of newly manufactured flippers. At the trailing edge, the material has lifted slightly above the desired level due to capillary effects.
- **Fig. S4. Fatigue test machine. (a)** Fatigue testing machine showing flipper in position **(b)** Controller showing cycle counter. **(c)** max cycle count of 922,154 cycles. **(d)** Showing twisting motion after 922,154 cycles and small trailing edge failure. **(e)** Close-up of the trailing edge failure.
- **Fig. S5. Test rig setup. (a)** Robot swimming past markers on a linear rail for obtaining swim speed data, followed by a camera on a linear rail running parallel with robot swim path **(b)** (1) Test rig setup for constrained operation (2) test rig setup for towing operation. **(c)** Pulley assembly. **(d)** Complete test rig assembly
- **Fig. S6. Test rig data from towing tests. (a)** The thrust produced by the turtle during tow testing **(b)** Swim speed data from tow test with an initial velocity of 0.6 m/s. **(c)** Drag produced by the turtle. The blue line represents the hydrodynamic drag force created by the turtle itself. The orange line shows the hydrodynamic drag forces with the addition of the friction forces from the test rig. **(d)** Data plot showing friction produced by linear rail assembly. Test produced without the robot attached. **(e)** Drag produced by towing robot at 0.45 m/s. Plot includes friction from linear rail assembly. Test produced with robot limbs held stationary

- **Fig. S7. Simplified Hardware Schematic for Turtle Controller**, Contains Particle Photon MCU, power regulator for MCU, servo signal and power headers with brownout protection.
- **Fig. S8. Test rig electrical system. (a)** Electrical system block diagram. **(b)** Electrical system test rig workstation setup, where 1 is the motor control system, 2 data acquisition system, 3 stepper motor and pulley assembly, 4 load cell assembly (drag). **(c)** Electrical system GUI.
- **Fig. S9. CFD results showing pressure contour plots on the robot with a focus around the robotic limb area**

3- Movie S1.mp4

This file provides a movie of a simulation of the robot's locomotor pattern.

4- Movie S2.mp4

This file provides a movie of the morphing and twisting flipper design.

5- Movie S3.mp4

This file provides a movie of the mechanical testing on the flipper design.

6- Movie S4.mp4

This file provides a movie of the robot performing various swimming tests

Any additional data sets or CAD files are available on reasonable request via direct contact to:

Dr Lorenzo Garcia

lorenzo.garcia@aut.ac.nz
BioDesign Lab
Auckland University of Technology
New Zealand
